# Data of feather recovering performance of birds and micro structure of pigeons’ feathers

**DOI:** 10.1016/j.dib.2019.105100

**Published:** 2020-01-07

**Authors:** Jing-Shan Zhao, Jiayue Zhang, Yuping Zhao, Zhaodong Zhang, Pascal Godefroit

**Affiliations:** a State Key Laboratory of Tribology, Department of Mechanical Engineering, Tsinghua University, PR China; b Royal Belgian Institute of Natural Sciences, Belgium

**Keywords:** Feather, Hierarchical vane structure, Self-healing, Flapping robot

## Abstract

Data is presented to explain why birds can recover their ruffled feather vanes by shaking wings and preening feathers with the beak [1]. Presented data includes the SEM microscopic images of rachis, barbs and barbules of pigeon's feather and the images recording the experiments of observing and mimicking the recovering performance of pigeons. Besides, based on the measurement and observation of the micro structure of feathers, the mechanical models of barbules were developed to better understand the wings performance. These high-quality images and models could be used for future research on feathers. Data helps to better understand the micro structure of feathers and the reason birds can fly. Data also support bioinspired mechanical structure development, especially for flapping robot development.

**Table:** Specifications Table

Subject	Bioengineering
Specific subject area	Bioinspired mechanical design
Type of data	Image
How data were acquired	1) Scanning electron microscope (JSM-7001F, Japan) operating at 3.0 KV acceleration voltage 2) High-speed camera with 180 frames per second
Data format	Raw and analysed data
Parameters for data collection	1) Images of scanning electron microscope of two contacted legs were obtained by a field-emission scanning electron microscope (JSM-7001F, Japan) operating at 3.0 KV acceleration voltage. We fixed a fraction of the feather by sticking it with conductive adhesive. Before SEM observation, the platform was coated with platinum. 2) Vanes of the 3rd, 4th, and 5th main flying feathers on the left and right wings of the pigeon were disrupted 3) The 5th main flying feather of the pigeon was chosen as the object of self-repair simulation experiment.
Description of data collection	1) Pictures of experiments were screenshotted from video recorded by high-speed camera 2) Images of feather micro structure were collected under SEM 3) Mechanical models of barbules are derived from the measurements and observations
Data source location	State Key laboratory of Tribology, Department of Mechanical Engineering, Tsinghua University, Beijing, P. R. China
Data accessibility	With the article Mendeley Data: https://doi.org/10.17632/kprk599r33.1
Related research article	Jing-Shan Zhao, Jiayue Zhang, Yuping Zhao, Zhaodong Zhang, Pascal Godefroit, Shaking the wings and preening feathers with the beak help a bird to recover its ruffled feather vane, Materials and Design, in press.

**Table:** 

**Value of the Data** • Reference dataset for research on feathers. SEM images clearly show the micro structure of feathers of pigeons. The images and models help to better understand the mechanism of how feathers capture air and have lift for flying. • Researchers who are interested in the mechanism of birds' flying and institute producing aircrafts will benefit from SEM images. Besides, researchers interested in bionic materials could benefit from the models of barbules. • SEM images and barbules models could be used to inspire bionic mechanical design. Understanding the structure of feathers, especially of barbules and micro hooklets helps to develop efficient flapping robot. The models will guide to make better biomimetic materials. • The data could be used for comparative studies for the feather structure of different birds, in order to find the most efficient flight mode.

## 1 Data description

**Fig. 1:**
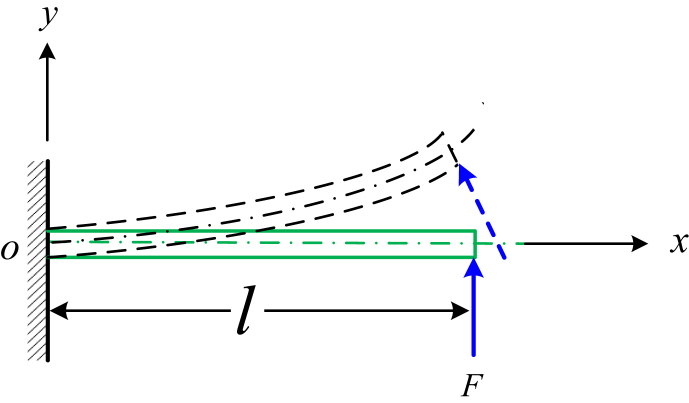
Bending vibration of a cantilevered beam. Under the action of the force F, the cantilevered beam will generate a deflection of $\delta =\frac{Fl^{3}}{3EI}$. After removing the force, the beam will output the bending vibration angle.

A series of pictures were captured by a high-speed camera with 180 frames per second to reveal the feather recovering performance of pigeons. There are basically two stages of this performance: shaking the wings and preening the ruffled feathers with the beak. The pictures are shown in the folder “Figure 1” in Mendeley Data: https://doi.org/10.17632/kprk599r33.1. The two stages are labelled with (A) and (B).

**Fig. 2:**
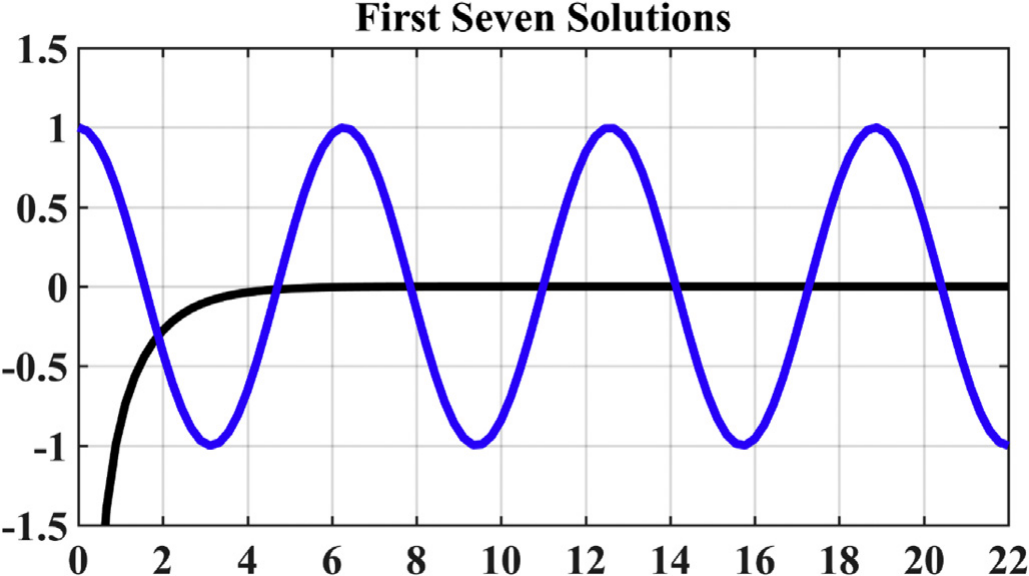
Solutions of equation cos(kl)=−sech(kl) for a cantilevered beam. Every cross point of the functions y=cos(kl) and y=−sech(kl) is one solution of the bending differential equation. There are infinite solutions in theory. Every solution corresponds to one primary mode. The leftmost cross point represents the first mode, and then the second, third and so on. Here we can get the first seven solutions which are illustrated by the abscissas of these 7 cross points.

To verify birds recover their messy feathers by shaking wings and preening with their beaks, an experiment mimicking this performance was done. The mimicking behavior was recorded with the same camera. The pictures are shown in the folder “Figure 2” in Mendeley Date: https://doi.org/10.17632/kprk599r33.1.

To be more specific, mechanical models of barbules were developed. The models are as follows:

The first primary vibration function:

(1) $$y\left(x,t\right)=C\left\{\cosh\left(1.875104\frac{x}{l}\right)-\cos\left(1.875104\frac{x}{l}\right)-0.7341\left[\sinh\left(1.875104\frac{x}{l}\right)-\sin\left(1.875104\frac{x}{l}\right)\right]\times \right\}\sin\left(\frac{1.875104^{2}}{l^{2}}\sqrt{\frac{EI_{z}}{\rho A}}t+\varphi \right)$$

The velocity at the cross section x is

(2) $$v\left(x,t\right)=\frac{1.875104^{2}C}{l^{2}}\sqrt{\frac{EI_{z}}{\rho A}}\left\{\cosh\left(1.875\frac{x}{l}\right)-\cos\left(1.875\frac{x}{l}\right)-0.7341\left[\sinh\left(1.875104\frac{x}{l}\right)-\sin\left(1.875104\frac{x}{l}\right)\right]\right\}\cos\left(\frac{1.875104^{2}}{l^{2}}\sqrt{\frac{EI_{z}}{\rho A}}t+\varphi \right)$$

The largest velocity is at x=l:

(3) $$v\left(l,t\right)=\frac{7.03203C}{l^{2}}\sqrt{\frac{EI_{z}}{\rho A}}\cos\left(\frac{1.875104^{2}}{l^{2}}\sqrt{\frac{EI_{z}}{\rho A}}t+\varphi \right)$$

The angular velocity can be approximately expressed as

(4) $$\omega \left(t\right)=\frac{7.03203C}{l^{3}}\sqrt{\frac{EI_{z}}{\rho A}}\cos\left(\frac{1.875104^{2}}{l^{2}}\sqrt{\frac{EI_{z}}{\rho A}}t+\varphi \right)$$

The largest value of the angular speed is

(5) $$\omega_{\max}=\frac{7.03203C}{l^{3}}\sqrt{\frac{EI_{z}}{\rho A}}$$

Suppose that there is a water drop attached to the end of the cantilever, the largest velocity it gets during the return motion is

(6) $$v_{\max}=\frac{7.03203C}{l^{2}}\sqrt{\frac{EI_{z}}{\rho A}}$$

The deflection of the barbule is a linear function of its length.

(7) $$\delta \left(x\right)=\int_{0}^{x}\frac{6ql^{3}dx}{Et^{3}}=\frac{6ql^{3}}{Et^{3}}x=\frac{6mgl^{3}}{EAt^{3}}x$$

The maximum deflection is $\delta_{\max}=\frac{6mgl^{4}}{EAt^{3}}$ occurring at the distal end of the barbule.

Every barb can be simplified as a larger cantilever the deflection of which has the same form:

(8) $$\delta \left(x\right)=\frac{6mgl_{B}^{3}}{EAt_{B}^{3}}x$$

where $l_{B}$ is the length of the barb and $t_{B}$ is the thickness of the barb.

## 2 Experimental design, materials, and methods

### 2.1 Materials and methods

#### 2.1.1 Image of scanning electron microscope of the feather

Images of scanning electron microscope of two contacted legs were obtained by a field-emission scanning electron microscope (JSM-7001F, Japan) operating at 3.0 KV acceleration voltage. We fixed a fraction of the feather by sticking it with conductive adhesive. Before SEM observation, the platform was coated with platinum.

#### 2.1.2 Observation of shaking and combing feathers of birds

We performed vanes disruption on the main flying feathers of the pigeons, the parrots and the white-eye birds, and then placed them in a pre-prepared glass room to observe the repair process of the disrupted flying feathers, while recording with a high-speed camera [1].

All of these three kinds of birds in the experiment were in good health and had normal behaviors, and the feathers of them were intact and orderly. We had established mutual trust during the long-term contacts with them, and they can naturally express their series of feather recovering behavior even when we observers were around.

The general experimental observation process of the pigeon includes:

• Firstly, some of the main flying feather vanes (of the 3rd, 4th, and 5th main flying feathers) on the left and right wings of the pigeon were disrupted. The state of the damage of the feather vanes could be summarized into two cases: one was that non-adjacent barbs formed “hook-to- groove” connection, or adjacent barbs formed misaligned “hook-to-groove” connection; the other was that adjacent barbs formed mismatch stack.
• We placed the pigeon into a cage and continued to observe the pigeon's performance, especially focusing on the recovery procedure of the damaged feathers. We found that the pigeon had a clear perception of the damage to its feathers. Immediately after we put the pigeon back into the cage, the pigeon would shake its body and wings, trying to recover the damaged feathers.
• Then the pigeon would use its beak to comb the feathers of the body surface in order. The pigeons showed different ways when combing the feathers. The pigeon's neck was very flexible and its head could be turned 180° or even larger angle. When combing the flying feathers on the wings, the head could hold the feathers from four directions, which were achieved through the torsion on both sides of the neck and upward or downward bending of the head. The pigeons made full use of the curvature of the upper beak and the flexibility of the neck, and used the beak to bite the feathers at different angles, which greatly increased the probability of the vanes being recovered.
• After repeated shaking and combing, the damaged vanes were recovered.

Based on the experimental observation of the pigeons, we investigated the parrots and the white-eye birds. Experiments proved that the feathers of the birds could be repaired from the messy state to the normal state by themselves. Although the recovering posture and frequency of the feathers of these three kinds of birds are different, the birds could eventually heal the feathers to an orderly natural state by shaking the feathers and combing the vanes with beak.

#### 2.1.3 Experiment of shaking the messy feather

We chose the 5th main flying feather of the pigeon as the object of self-repair simulation experiment. During the feather repair observation experiment of the pigeons, this 5th main flying feather was artificially injured, and the pigeon was also observed to comb this feather, which would help us to do comparative analysis. In order to ensure the integrity of the flying feather, the samples we selected in this experiment were obtained from living pigeons [1].

In this experiment we used a high-speed camera that could record 180 frames per second to record the whole feather combing process. We did a large area of damage treatment on the feather vanes, and the damage was much more serious than that the pigeons suffered in actual flight and life. We clamped the specimen feathers between two main feathers adjacent to it, and kept the narrow-edge vane of the feathers covering the wide-edge vane of the adjacent feathers. The spacing of two adjacent feathers was basically imitated the maximum unfolding state of the pigeon's wings during the feather shaking.

When the pigeons shook their feathers, they repeatedly flapped their wings, which not only made shaking of the feathers but also caused the collision between the adjacent feathers, thus the feathers were re-disrupted, and then recombined to the normal state. When we observed the feathering behavior of the pigeons, we found that the pigeons usually completed about 12 times of flapping action. Each flapping action included a complete cycle of the pigeon wings from the expansion to retract back to the initial state.

In this experiment, we held three feathers in the right hand, and then made a slap action on the back of the left hand, so that the feathers hit the back of the hand to produce vibration. After about 15–18 beats, most of the disrupted barbs were recovered.

#### 2.1.4 Experiment of combing the partially recover feather in shaking procedure

The pigeon used its beak to comb feathers, here we made a mimicry tool that was similar to the beak and simulated the combing experiment. We bended and sharpened the tweezer to get a similar curvature to the beak, the curvature of the upper beak of the pigeon was the key to the handmade process of the tweezer. When the pigeon combed the feathers, the curvature beak helped to hold the feather shaft, which could bend the feather shaft and reserve the elastic potential energy.

During the combing actions, the root of the feather shaft was held between the experimenter's left thumb and forefinger, while the right hand hold the cambered tweezer to comb the feather vane from the root to the tip. The head of the pigeon could be turned 180°, and it could be raised and lowered to achieve multi-angle and multiple combing to one flying feather, so that the flying feather would undergo barbs separation and recombination in various ways. We also imitated this multi-angle combing methods during the simulated combing experiment. In the stroke of combing the feathers with the imitation beak, the cambered tweezer played three roles, namely [1].

1) Separating the adjacent barbs that had partially unhooked.
2) Directly zipping the adjacent barbs that were originally slightly separated.
3) Pulling the feather shaft to bend and deform and store the elastic potential energy.

Among them, case 3) caused the feather shaft to be vibrated several times after being released, which promoted the recovery of the separated barbs. After one or several times of combing with the cambered tweezer, the feathers with a small number of barbs separated would be completely restored.

### 2.2 Derivation of the mechanical models of barbules

#### 2.2.1 Bending differential equation of a cantilevered beam

According to the scanning electronic microscopic microgram, the barbule can be simplified as a cantilevered beam (Fig. 1). Under the action of distributed airflow of high pressure, the shear force, the bending torque and the deflection of the beam can be calculated.

Every barb can be simplified as a cantilevered beam. The shear force is the lateral action of the tweezer. Suppose that the shear force is a constant value of *P* (Fig. 1). The bending moment of the beam at x is M(x)=P(l−x), the deflection is

$$\delta_{\max}=\int_{0}^{l}\frac{M\left(x\right)}{EI}\frac{\partial M\left(x\right)}{\partial x}dx=\int_{0}^{l}\frac{P{\left(l-x\right)}^{2}}{EI}dx=\frac{Pl^{3}}{3EI}$$

The differential equation of bending vibration of a cantilevered beam [2] is

(9) $$\frac{\partial^{2}}{\partial x^{2}}\left[EI\frac{\partial^{2}y\left(x,t\right)}{\partial x^{2}}\right]+\rho A\frac{\partial^{2}y\left(x,t\right)}{\partial t^{2}}=F$$

where E denotes the Young's elastic modulus of the material, I denotes the inertial moment of the section at x about the z-direction, ρ is the density of mass of the beam, A is its sectional area at x and F is a force to generate an initial deflection $y=\frac{Fl^{3}}{3EI}$. Therefore, we only need to discuss the homogeneous differential equation.

Suppose

(10) y(x,t)=Y(x)sin(pt+φ)

where Y(x) represents the shape function of bending vibration at x, p represents the frequency of a sinusoid vibration and φ represents the phase angle.

Also assume that the mass is uniformly distributed within the whole beam, the homogeneous differential equation (9) can be transformed into

(11) $$\frac{d^{4}Y}{dx^{4}}-\frac{\rho Ap^{2}}{EI_{z}}Y=0$$

The general solution of equation (11) is

(12) $$Y\left(x\right)=C_{1}\;\cosh\left(kx\right)+C_{2}\;\sinh\left(kx\right)+C_{3}\;\cos\left(kx\right)+C_{4}\;\sin\left(kx\right)$$

where $k=\sqrt[{4}]{\frac{\rho Ap^{2}}{EI_{z}}},\;C_{i}\left(i=1,2,3,4\right)$ can be determined by the initial boundary conditions of the beam. For the cantilevered beam (Fig. 1), the boundary conditions are

(13) {x=0,Y(0)=0,dY(0)dx=0,x=l,Y(l)=Fl33EI,dY2(l)dx2=0,dY3(l)dx3=0

Substituting Equation (13) into Equation (12) gains

(14) $$\left\{ \begin{array}{l} C_{2}=-C_{4}\\ C_{1}=-C_{3}\\ Y\left(l\right)=\frac{Fl^{3}}{3EI} \end{array}\right.$$

and

(15) $$\left\{ \begin{array}{l} C_{3}\left[\sin\left(kl\right)+\text{sinh}\left(kl\right)\right]+C_{4}\left[\cos\left(kx\right)+\text{cosh}\left(kl\right)\right]=0\\ C_{3}\left[\cos\left(kl\right)+\text{cosh}\left(kl\right)\right]-C_{4}\left[\sin\left(kx\right)-\text{sinh}\left(kl\right)\right]=0 \end{array}\right.$$

As $C_{3}$ and $C_{4}$ are nonzero constants, there must be

(16) $$\left|\begin{array}{cc} \sin\left(kl\right)+\text{sinh}\left(kl\right) & \cos\left(kl\right)+\text{cosh}\left(kl\right)\\ \cos\left(kl\right)+\text{cosh}\left(kl\right) & \sin\left(kl\right)-\text{sinh}\left(kl\right) \end{array}\right|=0$$

Therefore, we obtain from equation (8)

(17) cos(kl)=−sech(kl)

So we gain the solutions by drawing the graphs in the coordinate frame the abscissa of which is in the unit of kl. The abscissa of every cross point is one solution of equation (17). There are infinite solutions in theory. Each solution corresponds to one primary mode. However, only the first several solutions will be most easily excited in reality (Fig. 2 and Table 1).

**Table 1:** **First 6 solutions for the bending seta.** The cross points of the functions y=cos(kl) and y=−sech(kl) are the solution for the bending vibration of a cantilevered seta.

$k_{1}l$	$k_{2}l$	$k_{3}l$	$k_{4}l$	$k_{5}l$	$k_{6}l$
1.875104	4.694091	7.854757	10.995541	14.137168	17.278759

Therefore, we obtain that

(18) $$p_{n}=k_{n}^{2}\sqrt{\frac{EI_{z}}{\rho A}}$$

Substituting equation (14) into equation (12) yields the principal mode function:

(19) $$Y\left(x\right)=C_{4}\left\{\cosh\left(kx\right)-\cos\left(kx\right)+\frac{C_{3}}{C_{4}}\left[\sinh\left(kx\right)-\sin\left(kx\right)\right]\right\}$$

From any equation of (15) we can get the ratio of $\frac{C_{3}}{C_{4}}$. For example, we get $\frac{C_{3}}{C_{4}}=\frac{\sin\left(kl\right)-\sinh\left(kl\right)}{\cos\left(kl\right)+\cosh\left(kl\right)}$ when we select the second one in equation set (15). Then equation (19) will be simplified as

(20) $$Y\left(x\right)=C_{4}\left\{\cosh\left(kx\right)-\cos\left(kx\right)-\frac{\sinh\left(kl\right)-\sin\left(kl\right)}{\cosh\left(kl\right)+\cos\left(kl\right)}\left[\sinh\left(kx\right)-\sin\left(kx\right)\right]\right\}$$

As the first primary vibration is the case that is excited by the cantilevered force, we let$k_{1}l=1.875104$ and then we immediately gain

(21) $$Y\left(x\right)=C_{4}\left\{\cosh\left(1.875104\frac{x}{l}\right)-\cos\left(1.875104\frac{x}{l}\right)-0.7341\left[\sinh\left(1.875104\frac{x}{l}\right)-\sin\left(1.875104\frac{x}{l}\right)\right]\right\}$$

From the third boundary condition in equation (14), we know that the deflection of the cantilevered beam is $Y\left(l\right)=2.000C_{4}=\frac{Fl^{3}}{3EI}$, we then obtain that

(22) $$C_{4}=\frac{Fl^{3}}{6.000EI}=C$$

Substituting equations (18), (21) into equation (10) yields the first primary vibration function:

(23) $$y\left(x,t\right)=C\left\{\cosh\left(1.875104\frac{x}{l}\right)-\cos\left(1.875104\frac{x}{l}\right)-0.7341\left[\sinh\left(1.875104\frac{x}{l}\right)-\sin\left(1.875104\frac{x}{l}\right)\right]\times \right\}\sin\left(\frac{1.875104^{2}}{l^{2}}\sqrt{\frac{EI_{z}}{\rho A}}t+\varphi \right)$$

So the velocity at the cross section x is

(24) $$v\left(x,t\right)=\frac{1.875104^{2}C}{l^{2}}\sqrt{\frac{EI_{z}}{\rho A}}\left\{\cosh\left(1.875\frac{x}{l}\right)-\cos\left(1.875\frac{x}{l}\right)-0.7341\left[\sinh\left(1.875104\frac{x}{l}\right)-\sin\left(1.875104\frac{x}{l}\right)\right]\right\}\cos\left(\frac{1.875104^{2}}{l^{2}}\sqrt{\frac{EI_{z}}{\rho A}}t+\varphi \right)$$

Of course there is the largest velocity at x=l:

(25) $$v\left(l,t\right)=\frac{7.03203C}{l^{2}}\sqrt{\frac{EI_{z}}{\rho A}}\cos\left(\frac{1.875104^{2}}{l^{2}}\sqrt{\frac{EI_{z}}{\rho A}}t+\varphi \right)$$

The vibration looks like an oscillation about the root of the cantilever. So the angular velocity can be approximately expressed as

(26) $$\omega \left(t\right)=\frac{7.03203C}{l^{3}}\sqrt{\frac{EI_{z}}{\rho A}}\cos\left(\frac{1.875104^{2}}{l^{2}}\sqrt{\frac{EI_{z}}{\rho A}}t+\varphi \right)$$

The largest value of the angular speed is

(27) $$\omega_{\max}=\frac{7.03203C}{l^{3}}\sqrt{\frac{EI_{z}}{\rho A}}$$

Suppose that there is a water drop attached to the end of the cantilever, the largest velocity it gets during the return motion is

(28) $$v_{\max}=\frac{7.03203C}{l^{2}}\sqrt{\frac{EI_{z}}{\rho A}}$$

#### 2.2.2 Deflection of a cantilevered beam under the distributed load

**Fig. 3:**
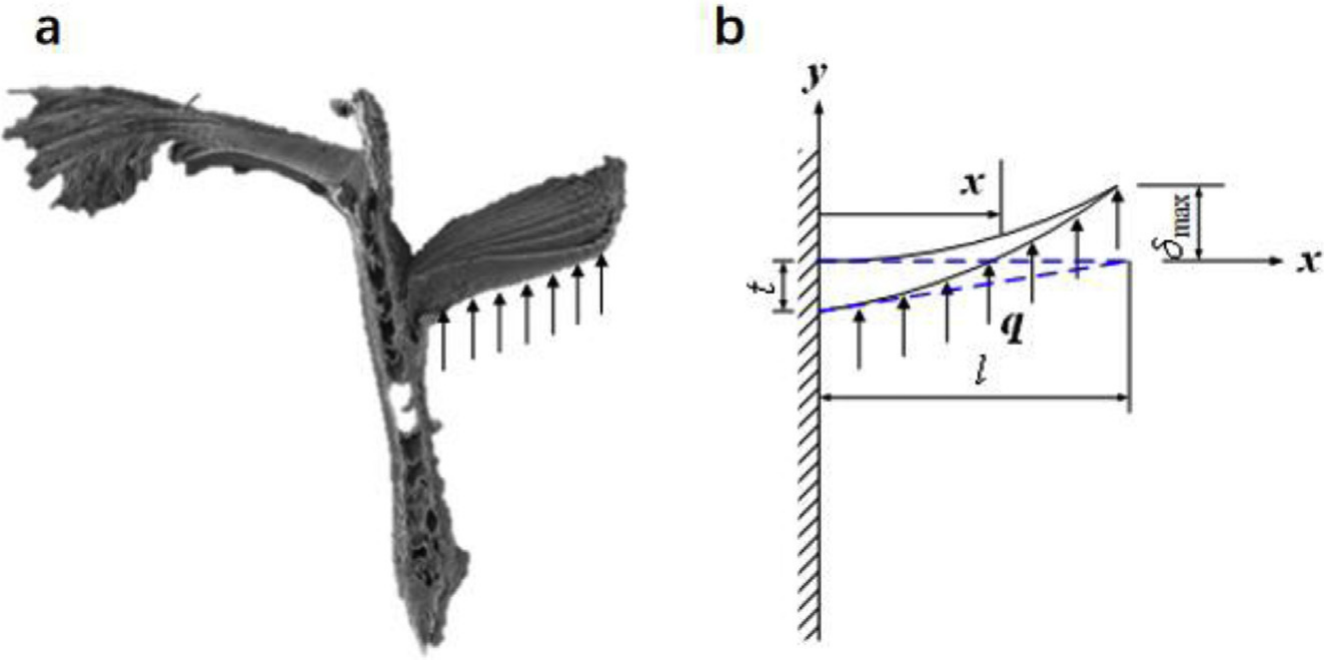
Cantilevered barbule and the air load distribution. (a) Every barbule on the barb is a cantilevered beam. Under the action of pressed airflow, the beam will generate deflection. (b**)** Deflection of a barbule under the distributed air pressure. The tip of the barbule has the largest deflection of $\frac{6mgl^{4}}{EAt^{3}}$.

Every barbule (Fig. 3A) can be simplified as a cantilevered beam. Under the action of distributed load of press air (Fig. 3B), the deflection of the tip barbule can analysed below.

Suppose that the density of the distributed air load is $q=\frac{mg}{A}$ where m is the total mass of the bird, g is the gravitational acceleration and A is the area of wings and body. Then the shear force is Q(x)=qw(l−x) and the bending moment is $M\left(x\right)=\int_{x}^{l}qw\left(y-x\right)dy=\frac{1}{2}qw{\left(l-x\right)}^{2}$ where w is the width of the beam. The slop of the tapered surface can be expressed as $k=\frac{t}{l}$ where t is the thickness of the beam at root and l is the length of the total beam, and therefore $I\left(x\right)=\frac{1}{12}w{\left(t-kx\right)}^{3}$. The deflection of the beam is

(29) $$\delta \left(x\right)=\int_{0}^{x}\frac{\frac{1}{2}qw{\left(l-x\right)}^{3}dx}{EI\left(x\right)}=\int_{0}^{x}\frac{6q{\left(l-x\right)}^{3}dx}{E{\left(t-kx\right)}^{3}}=\int_{0}^{x}\frac{6q{\left(l-x\right)}^{3}dx}{E{\left(t-\frac{t}{l}x\right)}^{3}}$$

Simplifying equation (29) yields

(30) $$\delta \left(x\right)=\int_{0}^{x}\frac{6ql^{3}dx}{Et^{3}}=\frac{6ql^{3}}{Et^{3}}x=\frac{6mgl^{3}}{EAt^{3}}x$$

Equation (30) indicates that the deflection of the barbule is a linear function of its length. The maximum deflection is $\delta_{\max}=\frac{6mgl^{4}}{EAt^{3}}$ occurring at the distal end of the barbule.

Similarly, every barb can be simplified as a larger cantilever the deflection of which has the same form.

(31) $$\delta \left(x\right)=\frac{6mgl_{B}^{3}}{EAt_{B}^{3}}x$$

where $l_{B}$ is the length of the barb and $t_{B}$ is the thickness of the barb.

**Fig. 4:**
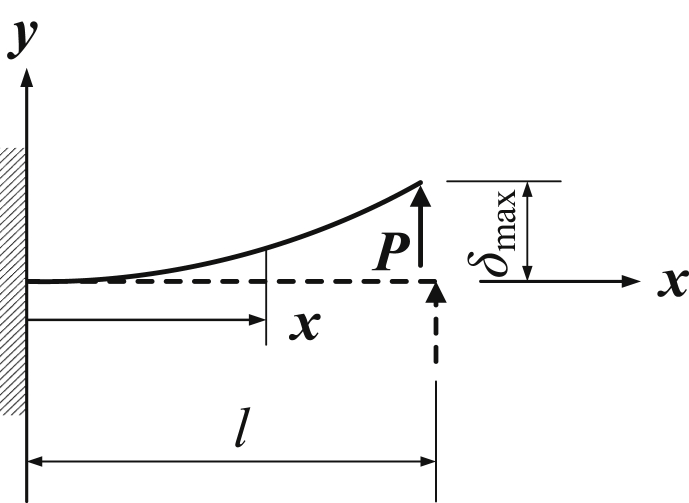
Bending vibration of a cantilevered beam. Under the action of the force F, the cantilevered beam will generate a deflection of $\delta =\frac{Fl^{3}}{3EI}$. After removing the force, the beam will output the restore to its initial position.

In the combing process, the barb will generate deflection (Fig. 4):

(32) $$\delta =\frac{Fl^{3}}{3EI}$$

where l is the length of the barb and I is the moment of inertia. Therefore, the barb will restore to its initial position after removing the combing force.
